# Non-Invasive Measurement of Frog Skin Reflectivity in High Spatial Resolution Using a Dual Hyperspectral Approach

**DOI:** 10.1371/journal.pone.0073234

**Published:** 2013-09-18

**Authors:** Francisco Pinto, Michael Mielewczik, Frank Liebisch, Achim Walter, Hartmut Greven, Uwe Rascher

**Affiliations:** 1 Institute of Bio- and Geosciences: Plant Sciences (IBG-2), Forschungszentrum Jülich GmbH, Jülich, Germany; 2 Institute of Agricultural Sciences, Eidgenössische Technische Hochschule (ETH) Zürich, Zürich, Switzerland; 3 Institut für Zellbiologie, Heinrich-Heine-Universität Düsseldorf, Düsseldorf, Germany; University of Gävle, Sweden

## Abstract

**Background:**

Most spectral data for the amphibian integument are limited to the visible spectrum of light and have been collected using point measurements with low spatial resolution. In the present study a dual camera setup consisting of two push broom hyperspectral imaging systems was employed, which produces reflectance images between 400 and 2500 nm with high spectral and spatial resolution and a high dynamic range.

**Methodology/Principal Findings:**

We briefly introduce the system and document the high efficiency of this technique analyzing exemplarily the spectral reflectivity of the integument of three arboreal anuran species (*Litoria caerulea*, *Agalychnis callidryas* and *Hyla arborea*), all of which appear green to the human eye. The imaging setup generates a high number of spectral bands within seconds and allows non-invasive characterization of spectral characteristics with relatively high working distance. Despite the comparatively uniform coloration, spectral reflectivity between 700 and 1100 nm differed markedly among the species. In contrast to *H. arborea*, *L. caerulea* and *A. callidryas* showed reflection in this range. For all three species, reflectivity above 1100 nm is primarily defined by water absorption. Furthermore, the high resolution allowed examining even small structures such as fingers and toes, which in *A. callidryas* showed an increased reflectivity in the near infrared part of the spectrum.

**Conclusion/Significance:**

Hyperspectral imaging was found to be a very useful alternative technique combining the spectral resolution of spectrometric measurements with a higher spatial resolution. In addition, we used Digital Infrared/Red-Edge Photography as new simple method to roughly determine the near infrared reflectivity of frog specimens in field, where hyperspectral imaging is typically difficult.

## Introduction

Coloration and color patterns of the integument of vertebrates vary considerably. Generally, the spectral properties of an integument are mainly defined by absorption and (diffuse) reflection occurring in its different layers, i.e. epidermis, dermis and subcutis, where differences in the pigmentation and in the structural organization of reflecting platelet layers lead to variations in the spectral reflectance [1]–[4]. The appearance of species in the visible range (VIS) from 400 to 700 nm is determined by the spectral reflectance characteristics of the integument and pigments, defining colors and patterns [Ibid].

This visible range is referred to human perception. However, visual perception is highly species-dependent and numerous vertebrate species can detect reflectance features outside the VIS, i.e., in the shorter wavelength (ultra-violet/UV) [5]–[7] and in the longer wavelength (far-red to infrared/IR) range [8]–[9]. UV-reflectivity and visibility as well as their biological relevance have been analyzed in numerous vertebrate taxa (for summary see 5–7), whereas studies on IR-reflectivity of the vertebrate integument are comparatively rare. Reflectivity of longer wavelengths has been demonstrated for some amphibians [10]–[13] and squamates [14]–[18]. However, the possible significance of this phenomenon is a matter of debate. In any case the near-infrared absorption contributes to heat load and thus may influence thermoregulation [12]–[19]. Furthermore it was suggested (despite the fact that near infrared vision has never convincingly been documented in any terrestrial animal) that increased reflectivity in the near infrared range (NIR) from 700–1400 nm may provide concealment from hypothetical predators or prey with extended vision in this range in environments of near high infrared reflectivity [10]–[12], [14], [20] as it is provided by green leaves [21].

Reflectivity of the integument of anurans shows a considerable variability in the NIR between species [10]–[12]. In some species NIR reflectivity is low, whereas in other species it can exceed reflectivity in the VIS [10]–[12], [22]–[24]. In the case of some anuran species, high (or low) reflectance in the NIR has been attributed to special structural properties of the dermal chromatophore unit [25]. Further, a possible association to an unusual red pterorhodin pigment, extracted for example from the melanophores of the dermal chromatophore unit of *Agalychnis dacnicolor* has been proposed [25]–[27]. Other elements present in the integument might have an influence on the spectral properties in these regions, for example water content or the number, composition and orientation of reflecting platelets.

Although the spectral reflectance of anuran species in the VIS was described in several studies, only few studies have measured the spectrum in NIR [28] and the short wave infrared (SWIR) part of the spectrum (1400–2500 nm) [29], [30]. There are numerous methods to measure optical properties of skins both ex vivo and in vivo, e.g. by either using single and double Ulbricht spheres or contact or non-contact spectrophotometers (for review see [31]). The former technique is highly invasive; the latter requires small working distances and thus is less suitable for jumpy creatures such as frogs. Both methods are limited to point measurements and therefore have a low spatial resolution. The low spatial resolution does not only refer to the limitation of point measurements in general, but also limits this approach to large structures. Multispectral or Hyperspectral imaging (HSI) is a state-of-the-art alternative to point spectroscopy and provides high spectral and spatial resolution.

HSI is a well-established method that has been used for example in remote sensing studies derived from air-borne and space-borne sensors (reviewed in [32], [33]), in quantification of plant functional traits [34], [35] and in medical investigations of the human skin, where it has been used to capture color based changes in concentration of chromophores such as oxyhemoglobin or deoxyhemoglobin [36].

In the present study we use for the first time a HSI system for in vivo measurements of frog skin reflectance. The system consists of two imaging spectrometers covering the range between 400–2500 nm. The spectrometers are characterized by a high spectral and spatial resolution and a high dynamic range with a relatively flexible working distance.

In addition we use digital IR/ red-edge photography as described recently [20] as a reference to visualize differences in the reflectance in the NIR range of the spectrum in a high spatial resolution.

### Investigated frog species

Investigations were performed on three green frog species. *Agalychnis callidryas*, the red-eyed tree frog, is an arboreal mesoamerican tropical hylid widely distributed in wet forest habitats from Mexico to Panama [37]. Adult specimens exhibit a bright, highly saturated green dorsal coloration with blue to orange striped patterns on cream white flanks. This pattern is highly variable among populations [38]–[40]. Exposed to direct sunlight *A. callidryas* might depict a whitish color [41]. With a length of 7 cm females are larger than males, which reach a size of 5 cm [42]. *A. callidryas* is strictly nocturnal hiding under leaves during the day [38].

*Hyla arborea*, (length 3 to 5 cm), the European tree frog, is distributed in Europe, where it preferably lives in lowland. Generally its dorsal surface is smooth and green. Depending on temperature and disposition, their color may change to various shades of brown, gray or specimens show a spotted pattern [43]. *H. arborea* is crepuscular and nocturnal spending the day on leaves often exposed to the sun [44].

White's tree frog *Litoria caerulea* is endemic to Australia and New Guinea and with reaching sizes of up to 10 cm it is the largest frog species examined by us [45]. Females are larger than males and both have smooth dorsal skin, which is colored olive green [45]. *L. caerulea* is able to change its color. Specimens are known to change colors and wine-red specimens are also known. The semiarboreal species is largely nocturnal [46].

## Materials and Methods

### Specimens and image acquisition

**Figure 1:**
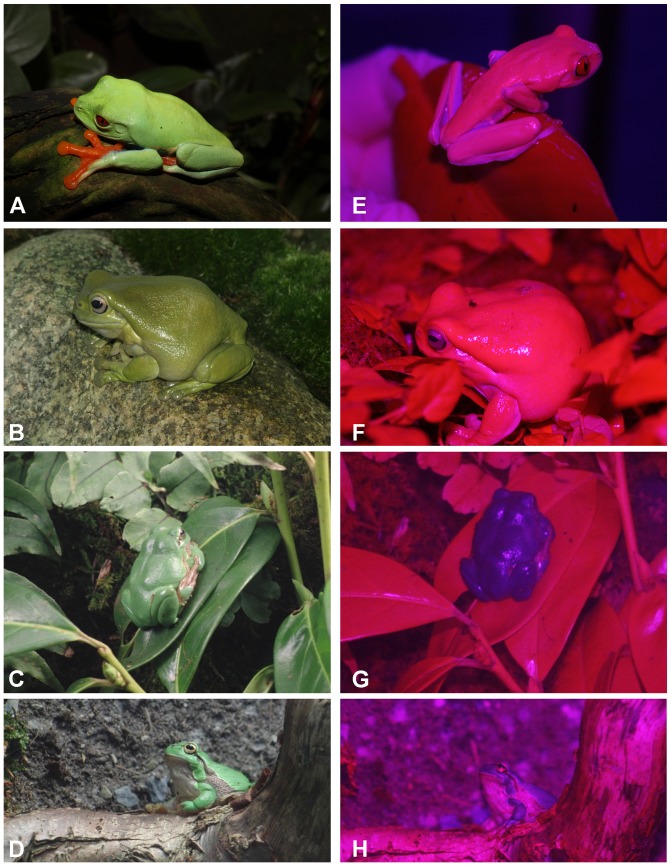
*Agalychnis callidryas* (A+E), *Litoria caerulea* (B+F) and *Hyla arborea* (C,D+G,H). Images acquired by digital color (left column) or red-edge photography (right column). Red-edge photographs (E–H) have been acquired by using a “two” channel camera (Red channel: Red+IR; Blue channel: blue) Canon EOS Digital Rebel XSi modified red-edge camera with removed hot-mirror substituted by maxmax.com, Canon 85 mm telephoto lens. (As the green pixels are missing (or very dark), overall images G and H were slightly brightness corrected). Color photographs (A–D): Image acquisition performed with a Canon EOS 400D camera.

**Figure 2:**
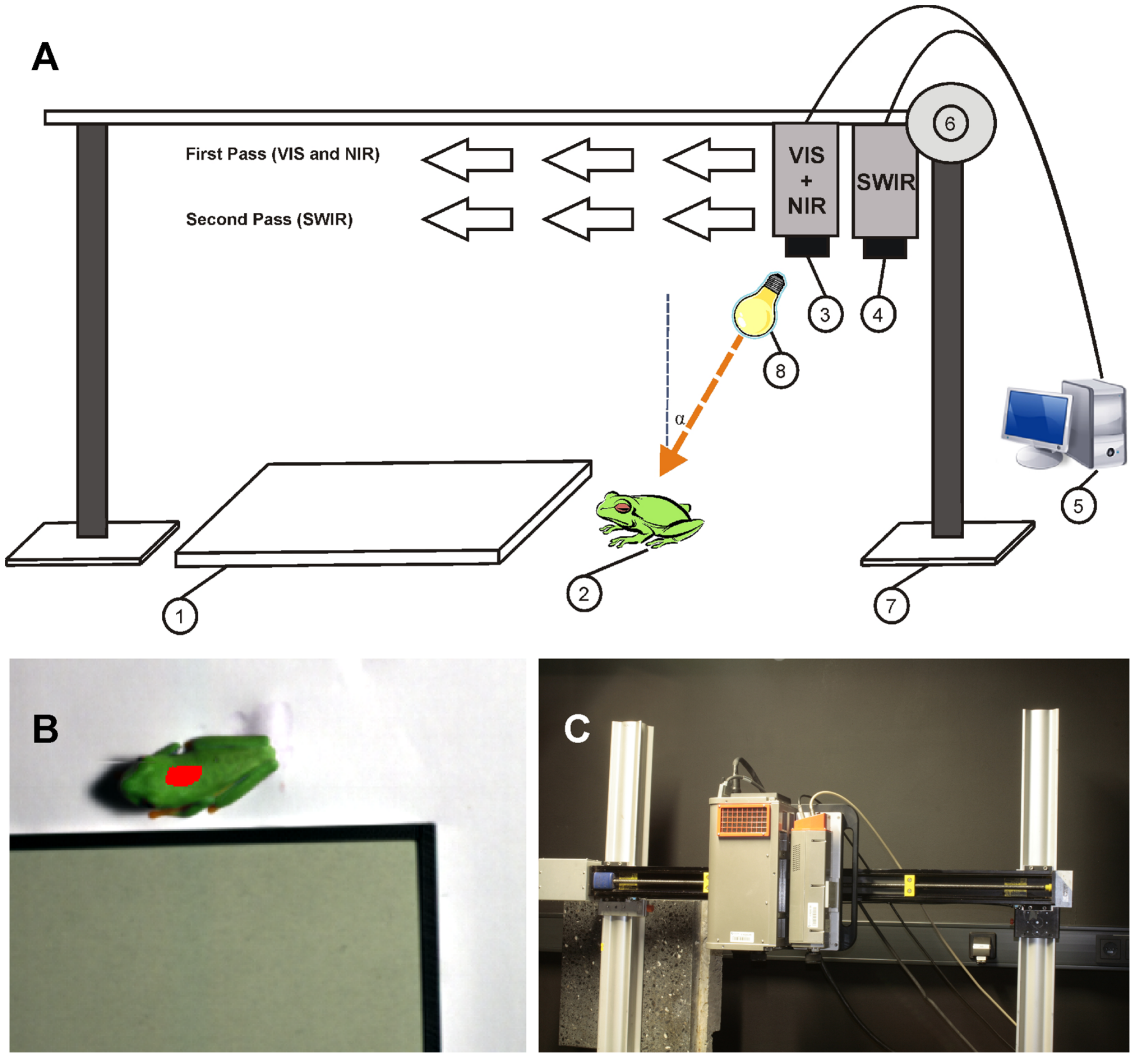
Hyperspectral imaging of three anuran species. (A) Hyperspectral imaging setup to acquire spectra and monochromatic images of a frog (2) and a reflectance standard (1) for wavelengths in the VIS, NIR and SWIR range of the spectrum. Images were acquired using two hyperspectral cameras (3 and 4) whereby the images were taken in a two pass setup. The complete setup is mounted on a solid aluminium stand (7). The first camera (3) allows to acquire a stack of hyper spectral images in the VIS and NIR (400 to 1000 nm). The second camera (4) acquires a stack of hyperspectral images in the SWIR (1000 to 2500 nm). Each camera can be driven by a stepping motor (6) along the axis of the metal bar seprateley during image acquisition in push-broom mode. Cameras and the moving stage were connected to a desktop computer (5), both controlling image acquisition and the moving-stage. Frogs (2) are imaged while sitting directly beside a 50% reflection standard (1). Illumination is provided using a halogen lamp (10) which ensured a sufficient amount light both in VIS, NIR and SWIR (8). (B) Exemplary area of interest used to calculate the reflectance spectra of *Agalychnis callidryas*. (C) Photo of the two hyperspectral cameras mounted on the moving stage.

Hyperspectral images of adults of three anuran species (*Agalychnis callidryas*, *Litoria caerulea* and *Hyla arborea*) that are characterized by a primarily green skin color (Fig. 1a–d) were taken in order to observe reflectance patterns in the VIS, NIR and SWIR part of the spectrum, particularly for selected areas of interest (AOI) (see Fig. 2b and Figures S1–S6). The study was not a field study, but was performed by imaging and photographing specimens that are maintained at the Aquazoo Löbbecke Museum in Düsseldorf, Germany. The animals were taken out of the terrarium for imaging and were individually transported within 30 min in small insulated plastic containers lined with moist filter paper from the Aquazoo-Löbbecke Museum Düsseldorf to the nearby Department of Biology of the University Düsseldorf, where the measurements took place. Here, hyperspectral measurements were taken in a conditioned lab. Animals were returned in their containers on the very same day to the Museum. We want to thank the Aquazoo Düsseldorf who supported us in our study. The Aquazoo is highly committed to nature conservation projects and itself participates in a number of international breeding and conservation projects of endangered species. As the animals used were no field animals, no special sampling was performed and permission to acquire images and photographs was granted to us by the Aquazoo. As the study is a strictly non-invasive restricted to “photography”, no approval was necessary.

Hyperspectral images were acquired in a darkened laboratory at room temperature and during image acquisition frogs were sitting inside a plastic box together with a 50% reflectance standard (Spectralon, Labsphere, North Sutton, New Hampshire, USA) (Fig. 2a,b). As it is known that some frog species might slightly change coloration to adjust to their local background, uniform reproducible white paper sheets were used to drape the inside of the plastic box. The distance between frog and hyperspectral cameras was 0.5 m. The scene was illuminated by a single lamp with a 500 W halogen bulb and a polished aluminum reflectance coating (Halogen Floodlight 500 W, Duewi, 63776 Duebris, Germany) placed at 30 cm distance to the frogs to provide homogeneous light in the range from 350–2500 nm. The incident angle of light was ca. 30° to the perpendicular relative to the reflectance standard and the investigated frog. A Schott glass in front of the lamp prevented the frogs to jump into it.

**Figure 3:**
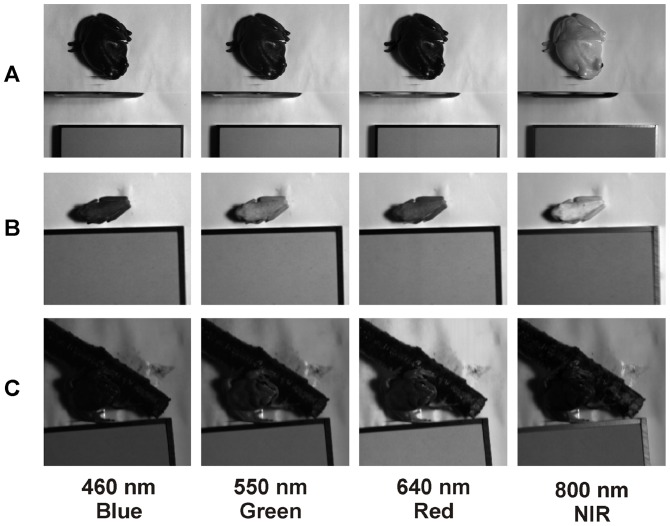
Hyperspectral imaging. Images of *Litoria caerulea* (A), *Agalychnis callidrya*s (B) and *Hyla arborea* (C) are shown for selected bands from the acquired hyperspectral cubes in the visible and NIR (460, 550, 640 and 800 nm).

**Figure 4:**
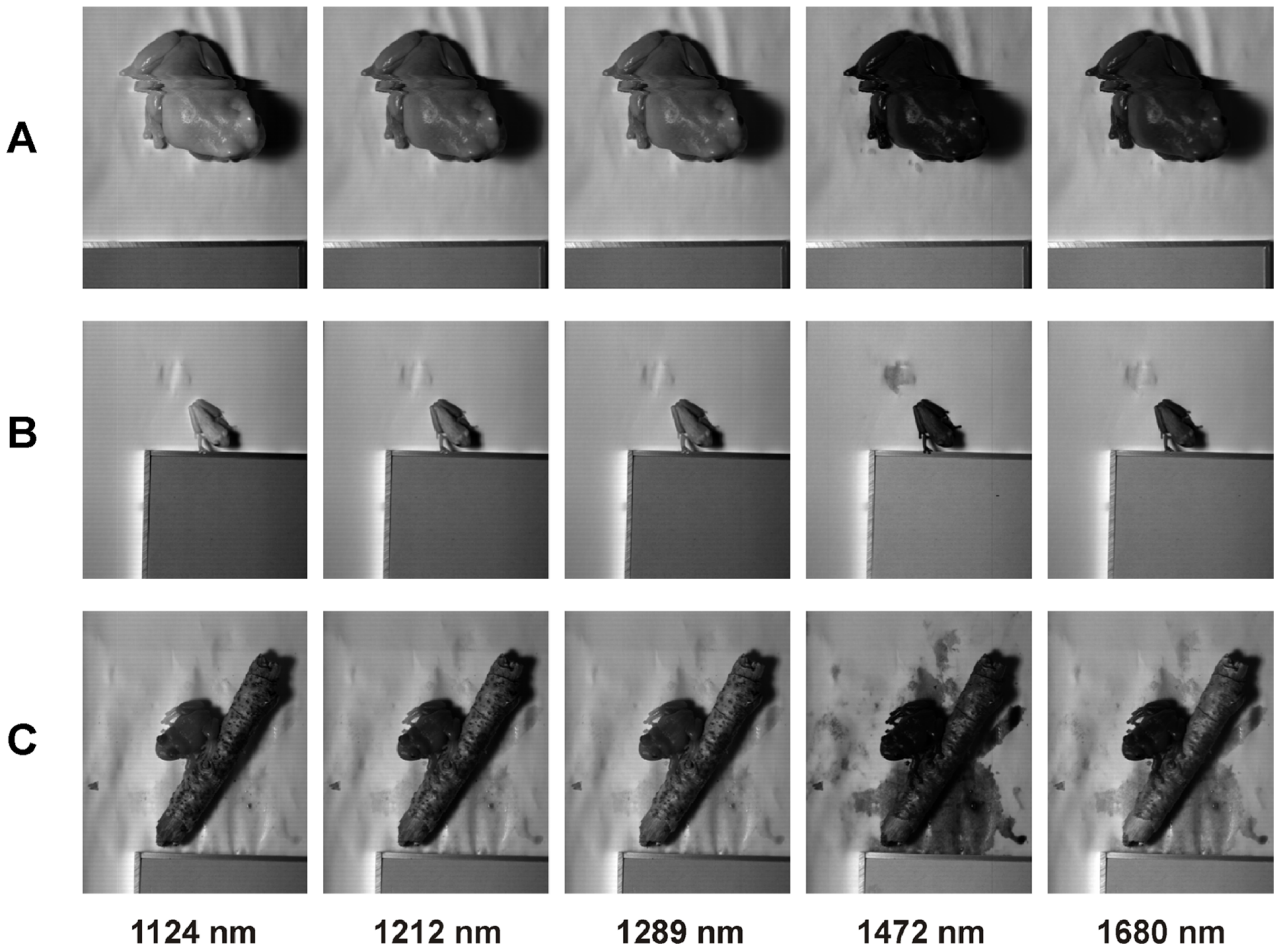
Hyperspectral imaging. Images of *Litoria caerulea* (A), *Agalychnis callidrya*s (B) and *Hyla arborea* (C) are shown for selected bands from the acquired hyperspectral cubes in the NIR and SWIR SWIR (1124, 1212, 1289, 1472 and 1680 nm).

During the hyperspectral image acquisition it was necessary that frogs sit motionless. Therefore, once the frogs were placed into the plastic box we gave them time to adapt to their new surroundings. While *A. callidryas* and *L. caerulea* showed quite an inactive behavior, *H. arborea* was very active, but became relaxed when a small stick of wood was provided as allurement (see Fig. 3, 4).

Acquisition of each hyperspectral data cube needed 2–4 min depending on the frog size and position within the box, plus a short time for focus adjustments. Animals were returned in their containers on the very same day to the Museum.

### Hyperspectral Imaging

Spatial distribution of the spectral reflectance of the frog skin was measured using two different imaging spectrometers, (1) a Spectral Camera PS V10E (Spectral Imaging Ltd. Oulu, Finland) and (2) a Spectral Camera SWIR (Spectral Imaging Ltd., Oulu, Finland) (Fig. 2c). The Spectral Camera PS V10E is an imaging spectrograph for the range of 400–1000 nm. It possesses a sensitive high speed interlaced CCD detector that provides spatio-spectral images of 1392×1040 pixels. It has a nominal spectral resolution of 2.72 nm Full width half maximum (FWHM) and a spectral sampling rate ranging from 0.63 to 5.06 nm depending on spectral binning. We worked using the full resolution of the camera, so neither spectral nor spatial binning was applied in our case. On the other hand, the SWIR camera acquires spectral information within the range of 970–2500 nm using a cooled, temperature stabilized MCT detector of 320×256 pixels. The spectral resolution is 10 nm (FWHM) with a spectral sampling rate of 6.3 nm. The distance between cameras and frogs (0.5 m) resulted in a spatial resolution of approximately 0.112 mm/pixel for the VIS/NIR camera and 0.4 mm/pixel for the SWIR camera.

Both devices are portable line scanning push broom imaging systems, i.e. the images are acquired while the camera moves over the object (see Fig. 2a). Thus, the camera acquires a 2D image, measuring in the X axis the spatial information across a line only (with specified length and small but finite width) and in the Y axis the spectral information (wavelength and intensity) for each point (pixel) of the X axis. Each camera has a fore optics that images a column of data on to a horizontal slit of 30 µm width at the entrance of the spectrometer. Light is spectrally spread in the Y axis by a diffraction grating and projected on the detector. For the PS camera a lens of 23 mm f/2.4 C-mount SP-OLE23 (Specim Ltd., Oulo, Finland) was used, while a 30 mm f/2.0 C-mount SP-OLES30 lens (Specim Ltd., Oulo, Finland) was used for the SWIR Camera. The second spatial dimension of the images is then generated by sequentially recording line images while the camera moves along a linear scanning bar. The spatial resolution in this second spatial dimension is defined by the scanning speed and the frequency of image acquisition. Both cameras were driven along a 1 m scanning stage (BiSlideTM 40′′, Velmex Inc. Bloomfield, NY, USA) mounted perpendicular to solid metal stands (X 95 profile system, Linos Photonics, Göttingen, Germany). The cameras and the scanning bar were controlled using the SpectralDAQ Software (Specim, Oulo, Finland).

**Figure 5:**
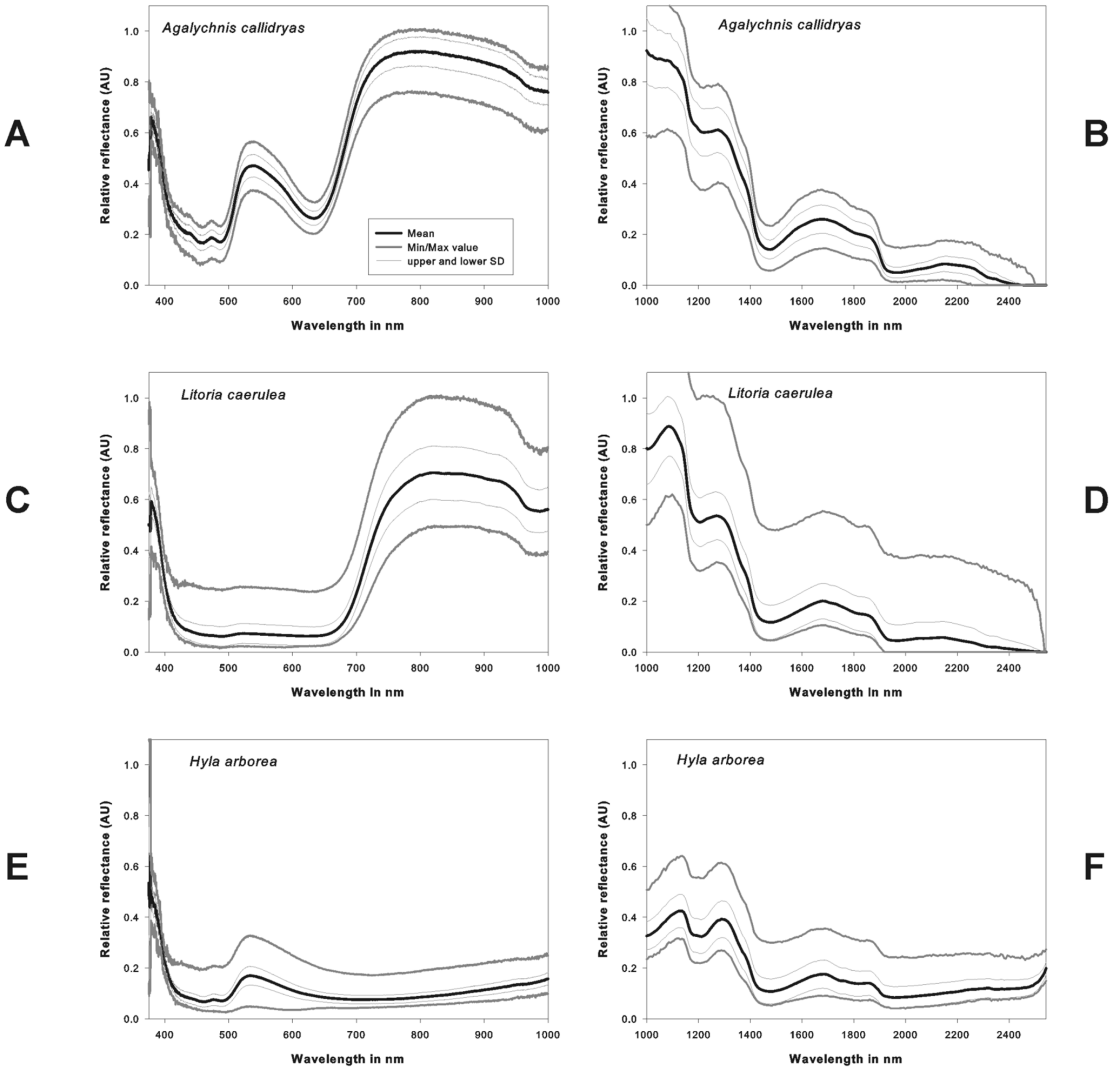
Spectral reflectance of dorsal coloration as a function of wavelength for a+b) *Agalychnis callidryias*, c+d) *Litoria caerulea* and e+f) *Hyla arborea*. Spectra were calculated from HSD data cubes from selected regions of interest. Shown are both reflectance in a+c+e) from 400 to 1000 nm (VIS and NIR) and b+d+f) in the range from 1000–2500 nm (SWIR). The VIS/NIR spectrum was calculated for a homogeneous area of dorsal skin from 4794, 246 and 443 pixels for *Litoria caerulea*, *Agalychnis callidryas* and *H. arborea* respectively. The SWIR spectrum was calculated for a homogeneous area of dorsal skin from 980, 362 and 273 pixels.

**Figure 6:**
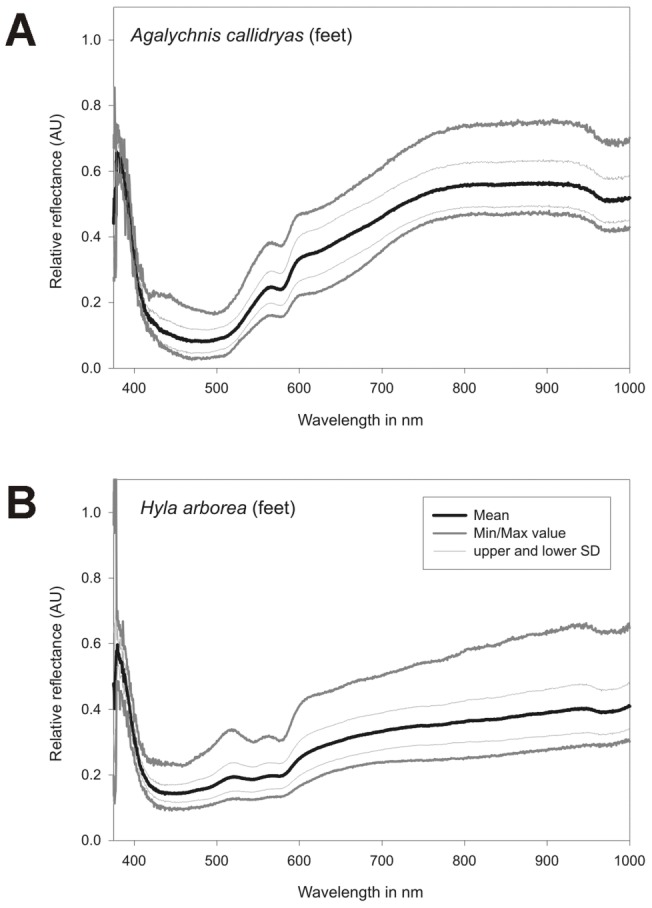
Spectral reflectance as a function of wavelength for toes of a) *A. callidryas* and b) *H. arborea*. Spectra were calculated from HSD data cubes from selected regions of interest. In both species additional characteristic absorption bands were found in the region of 520 to 580/NIR spectrum was calculated for a homogeneous area of interest consisting of 54 (*Agalychnis callidryas*) and 223 pixels *(Hyla arborea)*.

As a pre-processing step, hyperspectral data was linearly corrected using a pre-acquired dark image in order to remove the instrument noise. Then reflectance was calculated by normalizing to the 50% calibrated reflectance standard (Spectralon, Labsphere Inc., NH, USA) located near the frogs in each image. All pre- and post-processing-procedures of images were performed using the software ENVI (ITT VIS, Boulder, CO, USA). Comparing the spectral reflectance of the frogs, specific wavelength bands showing the most representative differences and features were selected. For each chosen wavelength band a gray scale image was produced using the same stretching thresholds in all frogs (see Fig. 3, 4). Brighter pixels represent higher reflectance for the specific wavelength. We subjectively selected representative wavelengths to illustrate spectral features of the species. Reflectance spectra (see Fig. 5, 6) were eventually calculated from processed hyperspectral data for the selected areas of interest (see Fig. S1–S6).

### Digital Infrared and Red-Edge Photography

**Figure 7:**
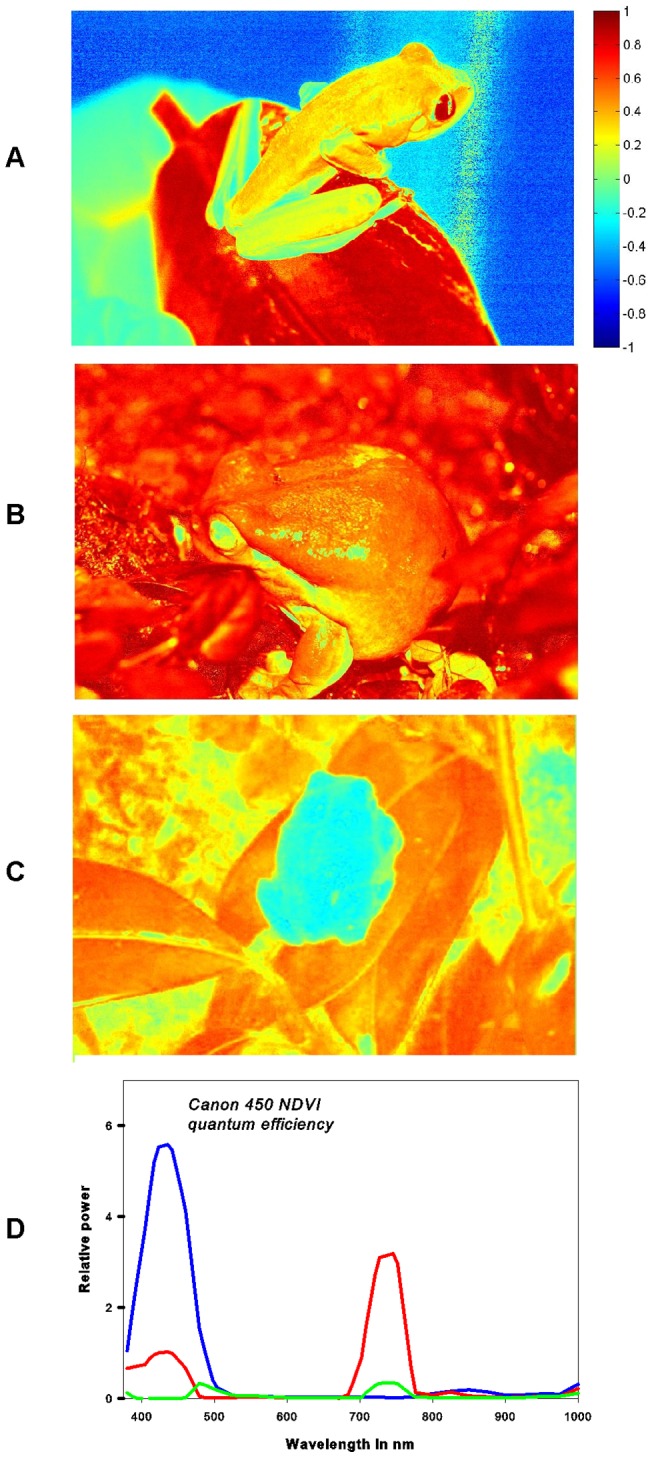
Normalized red-edge images of (A) *Agalychnis callidryas*, (B) *Litoria caerulea* and (C) *Hyla arborea* using a pseudo-coloration color scheme. The color index is given in figure(see equation 1+2). Raw images were acquired using a modifed Canon EOS Rebel XSi CCD camera which a optimized CCD sensitivity (D), in which the red channel is limited to the red-edge range of the spectrum (based on data provided by maxmax.com).



For infrared photography we used a Canon EOS Rebel XSi DSLR camera (12.2 megapixels), which was modified for red-edge photography (www.maxmax.com/vegetation_stress.htm), allowing indirect false color infrared imaging. This camera system has recently been used to differentiate between zoological specimens showing high and low NIR reflectance [20] and was described to be useful also in studies of vegetation cover [47]. In this camera, sensitivity of the red channel is shifted to the “red-edge” (670–770 nm), which is the spectral range of strongly increasing reflectivity in vegetation. The blue channel acquires light of short wavelength (370–480 nm) as a reference of light reflected in the visible part of the spectrum, while the green channel is omitted by filtering (for sensor sensitivity details see Fig. 7d). In vegetation studies NDVI images are generally calculated from Red and NIR channels applying equation (1) to every pixel of an image:

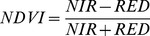



For the modified red-edge camera a deviant version of this equation was used to calculate normalized red-edge images (Fig. 7a–c) from the unprocessed RAW images (see Fig. 1e–h):
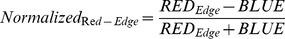



Color photographs were taken as comparison using standard digital consumer cameras.

## Results

### Hyperspectral measurements

The three investigated species showed generally a green coloration (Fig. 1). However, their spectral reflection properties were clearly different. In accordance with its coloration the dorsal skin of *Hyla arborea* showed a maximum peak in its VIS reflection spectrum with no marked increase in the NIR region (Fig. 5e). This can also be observed in the gray scale images, where at 800 nm the body of the frog is comparatively darker than at 550 nm (Fig. 3e). The reflection increased markedly only in the SWIR part range of the spectrum at 1000 to 1100 nm, showing absorption bands at 1200, 1450 and around 1900 nm (Fig. 5f). Reduced reflection in the NIR was also found in the unprocessed (Fig. 1g,h) and processed digital red-edge based (Fig. 7c) images. The arboreal tree-frogs green backside therefore was clearly outstanding in the NIR images compared to leaves on which they were sitting.

In contrast, *Agalychnis callidryas* and *Litoria caerulea* showed a remarkable increase in reflection in the NIR starting around 700 nm and remaining high until wavelengths of 1100 nm (Fig. 5a–d). A generally increased NIR reflection was also found in unprocessed (Fig. 1e, f) and processed (Fig. 7a,b) digital red-edge based as well as in digital b/w infrared photography (data not shown, but see Fig. 3, 4). Clear absorption bands in SWIR were detected in both species at 1200, 1450 and 1900 nm comparable to those found in *H. arborea* (Fig. 5b,d). Both species showed a much higher spectral-wide albedo compared to *H. arborea*. This is primarily due to the reduced reflection in the NIR (from 700 to 1000 nm). In the visible part of the spectrum *A. callidryas* showed a clear reflection peak in the green at 550 nm (Fig. 5a), whereas this feature was not observable in *L. caerulea*, which is in accordance with its grayish-green appearance. Interestingly, the spectral reflectance obtained from the orange toes of *A. callidryas* did not only show an increase in reflection around the orange part of the visible spectrum, but remained high up to 1000 nm (Fig. 6a). Reflectivity in the brownish toes of *H. arborea* was found to be relatively higher in NIR compared to that found in the VIS part of the spectrum (Fig. 6b). In both species such small scale analysis revealed additional minor absorption bands in the visible range of the spectrum. For instance, in toes of *A. callidryas* an additional absorption band was found around 570 nm (Fig. 6a), whilst two additional characteristic absorption peaks were found in the toes of *Hyla arborea* at 540 and 570 nm (Fig. 6b).

### Red-edge photography

Normalized red-edge images that were acquired with the modified two channel camera clearly show differences in the total reflectance of skins in the different species investigated. Both *Agalychnis callidryas* and *Litoria caerulea* show a very high near-infrared reflectance and thus appear reddish in the acquired photographs (Fig. 1). In contrast images acquired of *Hyla arborea* appear blue, due to the much lower reflectance in the NIR infrared range of the spectrum. The total NIR reflectance of *A. callidryas* and *L. caerulea* is generally comparable to that of green leaves and vegetation, yet differences become more pronounced if the acquired images are processed by the given normalized red-edge equation (eq. 2) shown in pseudo-color representation (Fig. 7).

## Discussion

### Technical notes – HSI

We have shown that HSI primarily developed for remote sensing and often applied for vegetation and stress monitoring in plants [34] can be used to measure skin reflectance of anurans. This technique is superior to previously used methods such as infrared photography and classical spectrophotometry as it combines the high spectral resolution of classical spectral monitoring with an improved spatial resolution, previously obtained only with color and infrared photography. Therefore, advantages of the herein used system are (1) monitoring of reflectance in a high spatial resolution, (2) covering of an extended spectral range from 400–2500 nm by combining two HSI systems, (3) non-invasive handling of the items to be analyzed, (4) a large working distance allowing examination of jumpy animals such as frogs, and (5) easy quantification of variance in coloration in the skin of one individual. Furthermore, multispectral or hyperspectral imaging of “natural scenes” [48] can be combined with HSI of specimens.

Hyperspectral imaging often referred to as ‘imaging spectroscopy’ opens the possibility for non-invasive quantification of constituents and non-supervised feature extraction. In this communication we only demonstrated the potential by manual selection of single wavebands. Advanced methods that take the full spectral signature into account include Partial Least Square Regression (PLSR; [49]), supervised and un-supervised endmember selection and unmixing, continuous support vector machines [50], multi-block analysis [51] or simplex volume maximization [35]. These methods were shown to provide significantly more accurate results than using single wavebands or difference indices. Additionally, such methods can be used to determine spectral regions that encode specific traits and features and help the selection of appropriate wavelength region for quantification [52].

Recently HSI was used to investigate color matching between cuttlefish-camouflage, background coloration and color perception of possible predators [53]. Chiao et al. (l.c.) strongly emphasized the potential usage of HSI imaging to study coloration of biological specimens, being superior compared to classical photographic studies as the color space can be modeled in accordance to possible predators. Thus, such a technique can be used in studies on visual cues and perception, and their importance in intra- and inter-specific communications.

Contrary to photographic methods or point spectroscopy, HSI needs more time for each analysis, as both spectral and spatial information have to be recorded. Nevertheless, using an automated scanning setup, acquisition time could be reduced to less than one minute, which is fast enough to record data while the frogs remain still. Typically, we found that in the first minutes after transfer frogs remained calm, though this might depend on both species and individual specific behavior.

In general HSI of anuran species showed to work best with species that have a smooth skin and those that show a comparably high overall reflectivity. In addition, it is worth noting that HSI worked surprisingly well in observations of small scaled structures such as eyes, color patterns (data not shown) or minute feet of froglets, providing not only suitable spatial, but also spectral resolution for detection of minor absorption bands in small spatial structures. For this, AOIs have to be selected carefully avoiding shadowed regions.

### VIS and NIR Spectral reflectance and absorbance properties of the integument of the examined anurans

Generally, our findings confirm previous studies, which describe reflectivity in the visible and near infrared part of the spectrum in frogs. *Litoria caerulea* was the first anuran species for which increased infrared reflection has been shown by infrared photography [22], [54]. The spectral properties in the visible and NIR part of the spectrum reported here for this species closely resemble those found in several other *Litoria* spp. [12], [27], whereas no increased infrared reflectivity was reported for *L. aurea* [27]. The relative small reflectance in the VIS found in *L. caerulea* is mostly related to a grayish-green color of the specimen during the measurements. Similar effects have been also reported in *Litoria infrafrenata* [12].

The spectral properties in the VIS and NIR part of the spectrum in *Agalychnis callidryas*, with a clear peak of reflectance at 550 nm and an increased NIR reflectivity from 700 nm to 1100 nm, closely resemble data found by Emerson et al. [12]. The increased infrared reflectivity was also detected in the normalized red-edge and b/w infrared photographs, resembling those reported previously by color infrared photography [10], [11]. High absorption between 400 to 500 nm and at ca. 650 nm was found in *A. callidryas*. Both features are related to the high pigment absorption in those regions.

Concerning the skin of *Hyla arborea*, and the related species *Hyla cinerea*, spectral reflectivity has been studied only in the visible spectrum [43], [55]–[59], but to our knowledge no spectral investigations extending into the NIR and SWIR spectral ranges have been performed previously. Our hyperspectral images clearly show high reflection at a wavelength of ca. 540 nm, which is in accordance to what was shown in previous articles cited above. On the other hand, no increased reflectivity in the red-edge or IR could be detected. Lack of increased infrared reflectivity is also obvious in the unprocessed and processed photographs acquired with the red-edge camera. Unprocessed red-edge photographs clearly render *H. arborea* blue when photographed sitting on a green plant leaf which is depicted in blood-red due to its own high infrared reflectivity. In this regard NIR reflectance properties of *H. arborea* closely resemble those of *H. cinerea* and other tree frogs studied by means of spectroscopy and color infrared [10], [12], [27].

The three anurans studied herein are arboreal species. The integument of one species, *H. arborea*, does not show NIR-reflectivity, whereas the increased NIR reflection of *L. caerulea* and *A. callidryas* markedly resemble those of plants investigated using the same setup (see 34). However, NIR reflectivity in the two latter species is significantly higher than that of green leaves, which typically reflect 40 to 50% [60], [61] of the incoming light (see Fig. 5), and is much higher than that of brownish soil.

In general differences in the refractive indices of different media arranged in multilayers may be responsible for increased reflectivity in the NIR [62]. Stacked media of different refractive indices can be found in the iridophores [63], [64]. Kobelt and Linsenmair [3] showed that platelets of the reed frog *Hyperolius viridiflavus* have a refractive index of 1.81 (compared to a 1.34 refractive index of the cytoplasm) with a calculated maximum of reflectivity occurring in the NIR, which could explain the increased reflectivity in the NIR compared to the visible part of the spectrum [3], [28]. In the examined anurans the structural basis for infrared reflectivity or its absence (in case of *H. arborea*) are not fully understood. Some investigations suggested that certain pigments as pterorhodin or even biliverdin might be responsible for increased NIR reflectivity in anurans [25], [26]. Melanin in contrast is known to absorb in the NIR infrared and thus contribute to reduce near-infrared reflectivity.

Possible advantages of the increased NIR reflection in some arboreal tree-frogs have been attributed to camouflage protection against hypothetical predators with visual sensitivities shifted to longer wavelengths or even capable to see in the NIR [10], [11].

### SWIR reflectivity in anurans

Only a limited number of studies have been performed on skin reflectivity extended into the SWIR part of the spectrum in amphibians [29], [30], [65], [66]. Hitherto existing spectral investigation on arboreal tree-frogs [12], [27] with unusually high NIR reflectance did not extended into the SWIR.

In general for the three investigated species we found SWIR spectral reflectance absorption bands close to 1200, 1450 and 1900 nm. This is in agreement with previous studies of skin properties of anuran species [28], [66], [67]. The absorption maxima are typical for H_2_O absorption characteristics and are reported both for the human skin [68]–[70] and desert squamates [66].

### Perfusion and the near-infrared reflectivity of small scaled structures

Photo-spectrometric analysis of tiny extremities can reveal unique features related to the perfusion of toes and other small scaled structures and we found that HSI also works well for investigations of them. Those features could not have been determined by using point spectrographs-based non-imaging analysis. In the orange toes of *A. callidryas* we found an increase in reflectance towards the NIR. This spectral reflectance appears to be comparable to those found for human skin [69], [70]. Both show (1) a reflectance in the NIR region which is slightly higher compared to that in the visible range of the spectrum, and (2) a characteristic absorption band around 570 nm. This spectral feature closely resembles one of the characteristic absorption bands (577 nm) of oxyhemoglobin observed in photometrical studies of human skin [70]. However, additional absorption bands of oxyhemoglobin and deoxyhemoglobin at 430 nm and 555 nm shown by Zonios et al. [70] were not detected in our measurements. Reflectivity of the skin of brownish *H. arborea* feet showed also a general increase in reflectivity from visible to NIR, but to a lesser extent than *A. callidryas*. In this case, however, two additional characteristic absorption bands were observed at 540 and 570 nm, thus matching two of the oxyhemoglobin absorption bands described for human skin reflectivity [70]. Yet, absorption bands of hemoglobin or oxyhemoglobin at 540 and 570 nm have neither been explicitly noticed in this, nor in any previous study from dorsal coloration of anurans, though they match absorption characteristics of anuran hemoglobin [71].

### Technical notes – Red-edge based photography

Hyperspectral imaging is a powerful tool to study animal coloration. Nevertheless, Stevens et al. [72] noted that the main disadvantage of hyperspectral cameras is that they may be slow and that specimens have to be stationary during image acquisition, which turned out to be unproblematic in our experiments. Hyperspectral imaging allows monitoring of small scale spectral differences and can be easily extended to spectral ranges which are outside the range common consumer color cameras including the SWIR and NIR range of the spectrum. Color cameras in contrast are easily to handle in field [72].

One of the main spectral differences between the species investigated by us is their either extremely high or low reflectivity in the infrared. It has been shown earlier that analogue black and white [22], [54] and color infrared photography [10], [11] can be used to differentiate between species showing different reflectivity in the NIR. Nevertheless, analogue color IR films are not produced anymore and black and white analogue films include no reference to the information related to channels in the visible range. Nowadays, common color CCD and CMOS based consumer cameras do not cover the NIR/IR range and provide very limited amount of information in regard of the red-edge. However, recently a red-edge camera based on a modified Canon EOS became available, which is used for vegetation studies and may provide a modern alternative [47]. Technically the camera sensor records a red and blue channel, whereby the red channel is especially limited to the range of the red-edge (see Fig. 7). Unprocessed pictures of Fi 1e–h already allow differentiating between frog species showing a high infrared and low IR reflectivity. In vegetation and remote sensing studies images are often generated by calculating NDVI derived images by using eq. 1. The red-edge camera used by us can generate comparable normalized indices that use the reflectance in the red-edge region (see Fig. 7a–c) using eq. 2. However, high blue reflectivity, as apparent for example in *A. callidryas* (see Fig. 1e, 7a) makes it difficult to simply relate the IR reflectivity of the frog specimens to vegetation reflectance that is driven by chlorophyll absorption. Yet such normalized indices can be used to segment green specimens with a low IR-reflectance such as *H. arborea* from green backgrounds having a high NIR-reflectance (i.e. leaves) (see Fig. 7c).

Overall we think that red-edge based imaging as recently described [20] is a simple but effective alternative to classical analogue or digital infrared photography that can be used in field supporting standard color photography in studies of animal coloration.

### Conclusions and future prospects

Our study shows the advantages of using of hyperspectral imaging and red-edge based photography for the spatial characterization of spectral reflectance of anuran integuments in the VIS- NIR and SWIR range of the solar spectrum. These advantages are: i) non-invasive and non-contact measurement, which make it suitable for in-vivo experiments, ii) use of spatial information to find changes within the same specimen or differences with the background and the information in the short wave infrared part of the spectrum and iii) the possibility to measure small spatial structures.

In particular, HSI has the potential to be used in studies investigating dynamic changes in spectral skin properties with respect to short-term color changes shown by many anurans [1]–[3], [56], [57], [73]–[75] and long-term changes induced among others by environmental cues [4], [28].

Though portability and light availability can be limitations, it might be also interesting to use HSI to investigate the background matching of anuran species in their natural habitats with local illumination and shadows. In this context it would be possible to apply spectral sensitivity models, if available, to the HSI data cubes, to assess the appearance of prey to the predators. This would allow for example to assess, whether visual pigments known to be sensitive for longer wavelengths, compared to the human sensitivity range, provide a general benefit in visual contrast ratios in the far red an near-infrared.

## Supporting Information

Figure S1 **Area of interest selected for spectral characterization of the skin of** ***Litorea cearulea*** **in the VIS/NIR part of the spectrum.** The average spectrum was calculated for a homogeneous area of dorsal skin (red region: 4794 pixels).
(PNG)

Figure S2 **Areas of interest selected for spectral characterization of the skin of** ***Agalychnis callidryas*** **in the VIS/NIR part of the spectrum.** The average spectrum was calculated for a homogeneous area of dorsal skin (red region: 246 pixels) and skin from the feet (green region: 54 pixels).
(PNG)

Figure S3 **Areas of interest selected for spectral characterization of the skin of** ***Hyla arborea*** **in the VIS/NIR part of the spectrum.** The average spectrum was calculated for a homogeneous area of dorsal skin (red region: 443 pixels) and skin from the feet (green region: 223 pixels).
(PNG)

Figure S4 **Area of interest selected for spectral characterization of the skin of** ***Litorea cearulea*** **in the SWIR part of the spectrum.** The average spectrum was calculated for a homogeneous area of dorsal skin (red region: 980 pixels).
(JPG)

Figure S5 **Areas of interest selected for spectral characterization of the skin of** ***Agalychnis callidryas*** **in the SWIR part of the spectrum.** The average spectrum was calculated for a homogeneous area of dorsal skin (red region: 362 pixels) and skin from the leg (green region: 54 pixels, not shown in this article).
(JPG)

Figure S6 **Areas of interest selected for spectral characterization of the skin and eyes of** ***Hyla arborea*** **in the SWIR part of the spectrum.** The average spectrum was calculated for a homogeneous area of dorsal skin (red region: 273 pixels) and from the eyes (green region: 135 pixels, not shown in this article).
(JPG)
