# Supplementary figures and images for: Non-Invasive Measurement of Frog Skin Reflectivity in High Spatial Resolution Using a Dual Hyperspectral Approach
Source: PLoS One. 2013 Sep 18;8(9):e73234. doi: 10.1371/journal.pone.0073234 (PMC3776832; doi:10.1371/journal.pone.0073234)

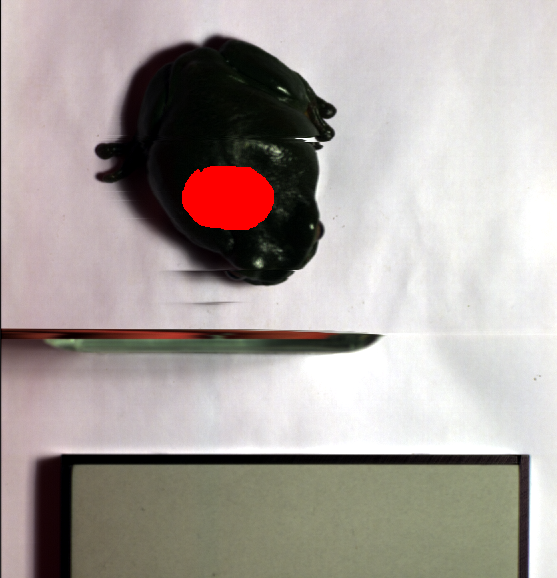

Supplement: Figure S1 — Area of interest selected for spectral characterization of the skin of Litorea cearulea in the VIS/NIR part of the spectrum. The average spectrum was calculated for a homogeneous area of dorsal skin (red region: 4794 pixels). (PNG) [file pone.0073234.s001.png]

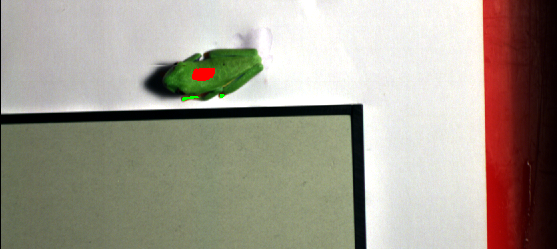

Supplement: Figure S2 — Areas of interest selected for spectral characterization of the skin of Agalychnis callidryas in the VIS/NIR part of the spectrum. The average spectrum was calculated for a homogeneous area of dorsal skin (red region: 246 pixels) and skin from the feet (green region: 54 pixels). (PNG) [file pone.0073234.s002.png]

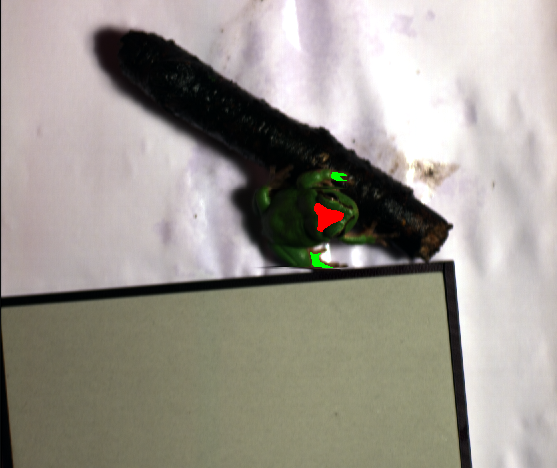

Supplement: Figure S3 — Areas of interest selected for spectral characterization of the skin of Hyla arborea in the VIS/NIR part of the spectrum. The average spectrum was calculated for a homogeneous area of dorsal skin (red region: 443 pixels) and skin from the feet (green region: 223 pixels). (PNG) [file pone.0073234.s003.png]

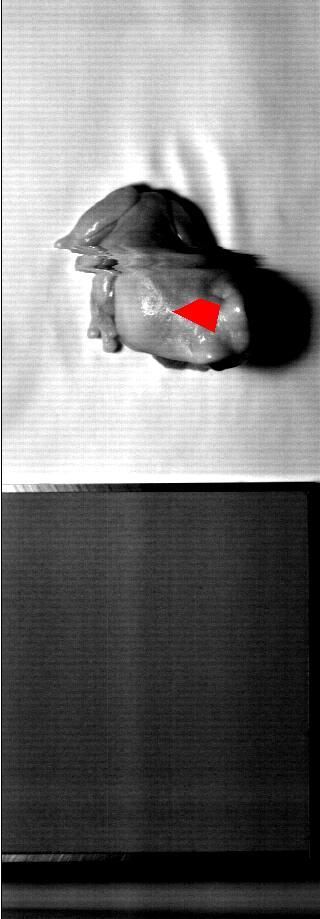

Supplement: Figure S4 — Area of interest selected for spectral characterization of the skin of Litorea cearulea in the SWIR part of the spectrum. The average spectrum was calculated for a homogeneous area of dorsal skin (red region: 980 pixels). (JPG) [file pone.0073234.s004.jpg]

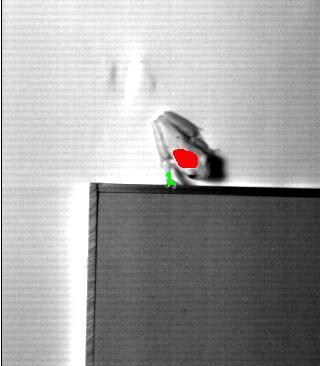

Supplement: Figure S5 — Areas of interest selected for spectral characterization of the skin of Agalychnis callidryas in the SWIR part of the spectrum. The average spectrum was calculated for a homogeneous area of dorsal skin (red region: 362 pixels) and skin from the leg (green region: 54 pixels, not shown in this article). (JPG) [file pone.0073234.s005.jpg]

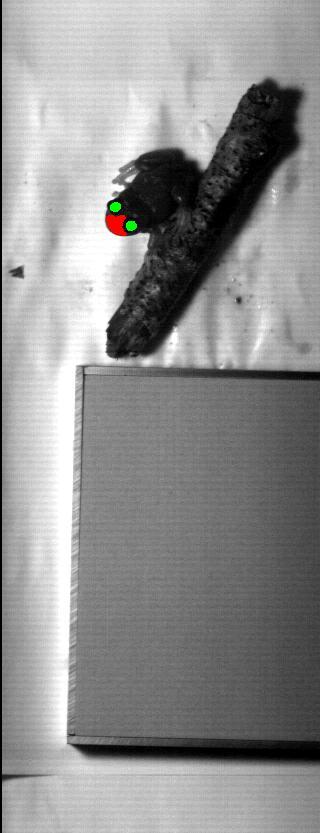

Supplement: Figure S6 — Areas of interest selected for spectral characterization of the skin and eyes of Hyla arborea in the SWIR part of the spectrum. The average spectrum was calculated for a homogeneous area of dorsal skin (red region: 273 pixels) and from the eyes (green region: 135 pixels, not shown in this article). (JPG) [file pone.0073234.s006.jpg]
